# PRISM, a Novel Visual Metaphor Measuring Personally Salient Appraisals, Attitudes and Decision-Making: Qualitative Evidence Synthesis

**DOI:** 10.1371/journal.pone.0156284

**Published:** 2016-05-23

**Authors:** Tom Sensky, Stefan Büchi

**Affiliations:** 1 Centre for Mental Health, Department of Medicine, Imperial College London, London, United Kingdom; 2 Clinic for Psychotherapy and Psychosomatics “Hohenegg”, Meilen, Switzerland; IRCCS Istituto Auxologico Italiano, ITALY

## Abstract

**Background:**

PRISM (the Pictorial Representation of Illness and Self Measure) is a novel, simple visual instrument. Its utility was initially discovered serendipitously, but has been validated as a quantitative measure of suffering. Recently, new applications for different purposes, even in non-health settings, have encouraged further exploration of how PRISM works, and how it might be applied. This review will summarise the results to date from applications of PRISM and propose a generic conceptualisation of how PRISM works which is consistent with all these applications.

**Methods:**

A systematic review, in the form of a qualitative evidence synthesis, was carried out of all available published data on PRISM.

**Results:**

Fifty-two publications were identified, with a total of 8254 participants. Facilitated by simple instructions, PRISM has been used with patient groups in a variety of settings and cultures. As a measure of suffering, PRISM has, with few exceptions, behaved as expected according to Eric Cassell’s seminal conceptualisation of suffering. PRISM has also been used to assess beliefs about or attitudes to stressful working conditions, interpersonal relations, alcohol consumption, and suicide, amongst others.

**Discussion:**

This review supports PRISM behaving as a visual metaphor of the relationship of objects (eg ‘my illness’) to a subject (eg ‘myself’) in a defined context (eg ‘my life at the moment’). As a visual metaphor, it is quick to complete and yields personally salient information. PRISM is likely to have wide applications in assessing beliefs, attitudes, and decision-making, because of its properties, and because it yields both quantitative and qualitative data. In medicine, it can serve as a generic patient-reported outcome measure. It can serve as a tool for representational guidance, can be applied to developing strategies visually, and is likely to have applications in coaching, psychological assessment and therapeutic interventions.

## Introduction

There are frequently circumstances in medicine and in the social sciences when it can be useful to gather personally salient attitudes or attributions from an individual or a group. Examples include patient health outcomes, health and environmental risk appraisal, attitudes to the workplace, and group appraisals of meetings or training sessions. Gathering and aggregating personally salient data can be difficult and time consuming. Nomothetic methods, for example generic questionnaires, can elicit information about known and/or frequent attitudes. However, such methods are very unlikely to access the full range of individual attitudes within any group. Idiographic methods such as personal narratives and interviews have the potential to elicit the full range of attitudes in any given group, but such methods can be difficult to apply in practice because they are time-consuming and because aggregating data from individual informants can be difficult [1].

The Pictorial Representation of Illness and Self Measure (PRISM) [2] is a novel and simple method of eliciting and aggregating personally salient information. PRISM was originally conceived as a simple assessment of how people coped with serious illness. However, initial pilot work demonstrated that PRISM was not measuring coping. Nevertheless, the pilot work did indicate that individuals responded to PRISM in a consistent manner. Quantitative and qualitative data demonstrated that, in its initial form, PRISM behaved exactly as expected of a measure of suffering [3,4]. According to Cassell’s seminal definition [5], suffering occurs when the individual perceives a serious threat to his or her Personhood, defined by people, things and qualities uniquely important to him or her. Among people who have serious illnesses, suffering is expected to be influenced more by the individual’s appraisal of the threats to Personhood than by the seriousness of the illness defined by healthcare specialists.

PRISM was not developed on a sound conceptual basis. Its utility as a measure of personal suffering was discovered serendipitously, but supported by validation studies. More recently, researchers, clinicians and people organising surveys have started applying PRISM in different ways (see ‘Other applications of PRISM‘, below). These novel and increasingly diverse applications of PRISM have highlighted the need for a wider review of PRISM, and a reappraisal of its broad conceptual basis.

Given these developments, the aim of this paper was to carry out a systematic review of publications which have reported data using PRISM, to produce a generic conceptualisation of how PRISM works.

## Methods

This study used qualitative evidence synthesis [6]. This involves comparing and integrating findings from relevant studies. It is a form of systematic review, aiming to identify themes or constructs from the papers studied, with the aim of providing an overarching narrative and, as in this case, developing a theory [6]. By choosing this method, our aim was to add to the understanding of PRISM as a measure of suffering but also to contribute to a broader generic conceptualisation of the PRISM task.

**Fig 1:**
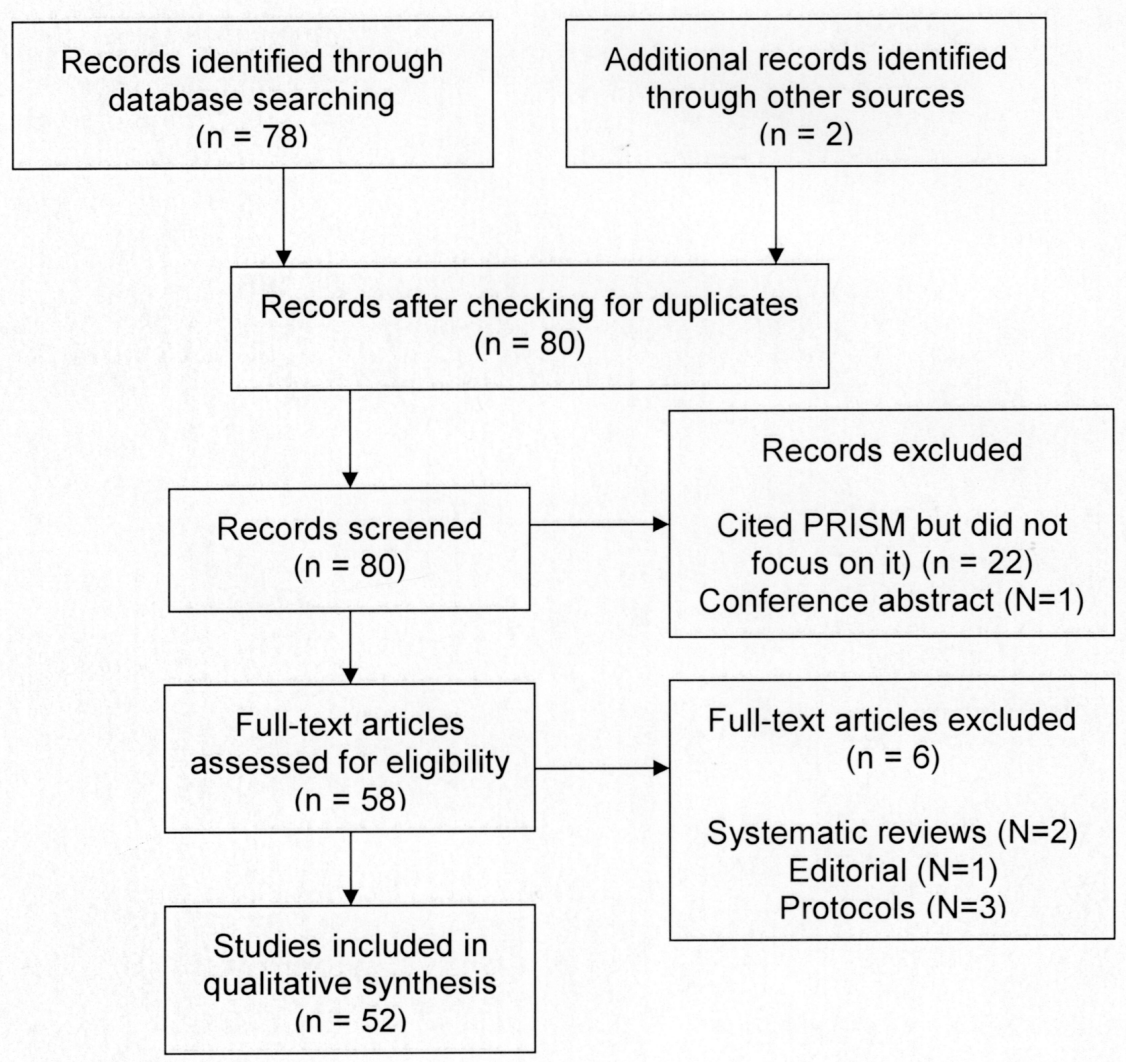
PRISMA flowchart.

Publications referring to PRISM were identified by retrieving papers which cited the original papers describing the PRISM and PRISM+ tasks [2–4], using Ovid Medline, Scopus, and the Web of Science Core Collection, supplemented by searches using Google Scholar. For example, in Scopus, the three papers above were searched for, then “View Cited by” was selected to generate the citations. Similarly, when the abstract of each paper was opened in Web of Science, this led to the Citation Network, giving the number of times the paper had been cited. Clicking on this yielded the citations themselves. This search identified 80 citations up to May 2015 (see Fig 1). These citations included two systematic reviews [7,8], one editorial [9] and three published protocols, without any data. Details of the search are shown in the PRISMA flowchart (see also S1 PRISMA 2009 Checklist and S1 PRISMA Checklist Notes).

Fifty two papers were identified which included data on PRISM, including a total of 8254 patients or participants (see S1 Appendix: Bibliography of papers reported data using PRISM). Details of the study samples, methodology and results were abstracted on to a spreadsheet, and reviewed independently by both authors. Patterns or trends were sought among these details, including the identification of anomalous or apparently disconfirming results [10]. While systematic reviews usually include appraisal of the quality of papers included in the review, this was not done in this instance. The main focus was on the PRISM task which has very simple instructions (see below), allowing it to be carried out consistently across studies. However, where a study yielded apparently anomalous results compared with other similar studies, the methods were examined to determine whether the anomalous results could be explained by how PRISM had been used.

## Results

The original PRISM task will first be described, then modifications to the original task. Some details will then be summarised regarding the general use of PRISM. Most of this information comes from the original studies, but has been supplemented with modifications and observations from later publications. Following this, results will then be highlighted from studies which have used PRISM to measure suffering, and then from studies reporting other applications of PRISM.

### The PRISM task

In the standard PRISM task, based on the original task to measure suffering [2,4], informants were shown a white A4-size metal board (297 x 210mm), with a fixed, yellow disk 7cm in diameter at the bottom right hand corner. Informants were instructed: “Please imagine that this white board is your life at the moment, and the yellow disk [point to the disk] is your ‘Self’.” Informants were then handed a red disk, 5cm in diameter, and told: “Now imagine this red disk is your illness. Where would you put the Illness to reflect its importance in your life at the moment?”. The main quantitative measure derived from PRISM is the distance between the centres of the Self and Illness Disks (termed the Self-Illness Separation, or SIS).

In all the studies carried out to date using PRISM, the vast majority of informants have understood these simple instructions, and placed the Illness disk on the PRISM board. As will be noted below, the resultant Self-Illness Separations were not random, but behaved as expected of a measure of suffering (the smaller SIS, the greater the person’s suffering). However, 5% or fewer informants have failed to understand the task according to the basic instructions [4,11–13]. These informants were shown a second, blue, disk, again 5cm in diameter. They were told: “Let’s say that this blue disk represents your work [some other specified aspect of life can be substituted]. If work is a very important part of your life at the moment, you might place the blue disk somewhere here [blue disk placed near the Self disk]. On the other hand, if work is not at all important in your life, you might place the Work disk here [place the blue disk in the corner diagonally opposite the Self disk ie as far as possible from the Self].” Informants were then asked again to place the Illness disk on the PRISM board.

Having placed the Illness disk on the PRISM board, informants were asked why they placed their Illness in the position they chose. In most instances, a succinct explanation was offered, which could be written down or audiotaped.

Some aspects of the initial design of the PRISM task were arbitrary, such as the colours of the Self and Illness disks, and their diameters. The decision to place the Self in the corner of one’s life, rather than at its centre, was a purely pragmatic one, to allow the greatest range of distances between Illness and Self. However, some researchers have placed the Self disk at the centre of the PRISM board, or changed the size of the board.

### The PRISM+ task

In this extension of the basic PRISM task, informants were asked, having completed the basic PRISM task, to place other disks (usually of the same size but in different colours) on the PRISM board, to assess other aspects of Personhood, such as the importance of work and/or leisure activities in the informant’s life. Personhood was the term used by Cassell to define the totality of people, things and qualities uniquely important to the individual [5,14]. In essence, the PRISM board allowed chosen aspects of Personhood to be mapped in terms of their importance. With several aspects of Personhood being considered, the standard design of the PRISM board forced informants to prioritise these aspects, because they could not all be placed at the same distance from the Self disk (unlike with a round PRISM board, with Self in the middle).

PRISM+ has been valuable as a component of clinical interventions for individual patients [3]. If, for example, a patient places on the PRISM board disks representing the illness, her children and her work, the clinician can ask not only why the patient placed the disks where she did, but also where she wants each of the disks to be, what can be done to move the disks to their preferred locations, and who can contribute to these moves (the patient, family supports, the clinician, other clinicians, etc). Patients often value having such a ‘visual summary’ of their present situation and how they wish things to change.

In some studies, the disk(s) other than the illness disk to be placed on the PRISM board have been defined and labelled by the researchers. In their study of people with chronic non-cancer pain, Kassardjian and colleagues asked their informants to locate on the PRISM board not only their pain, but also partner, family, work and recreational activities [15]. In another study of cancer survivors with severe fatigue, informants were asked to place two disks, representing their cancer and their fatigue, on the PRISM board [16]. In their study of victims of human rights violations, Stammel et al [17] used two disks representing the informants’ and the perpetrators’ cultural groups. In other studies, additional disks have not been labelled *a priori* by the researchers, but informants have been asked to label them with personally salient aspects of their lives [3,18]. Another approach has been to give the additional disks a generic label, such as ‘resources available to me’, and ask informants to give a specific label to each disk reflect their own resources [19–21].

### Modifications of the original task

The original PRISM task was intended to be introduced to the informant by a researcher. However, Rumpf and colleagues reported successful application of a paper-and-pencil version of PRISM, sent to informants for self-completion [22]. Other studies have used this paper-and-pencil version of PRISM, either self-administered [23–26] or with instructions from a researcher [27]. One of these studies asked informants not only to indicate the place of their illness in their life, but also to indicate where someone caring for them would place the illness [25].

Most researchers have used a PRISM board which is either rectangular, following the original design, or square [22], with the ‘Self’ disk in one corner. However, some studies have made the PRISM board circular, with the Self disk placed at the centre of the board [28,29]. Vingerhoets and colleagues have made a further modification to the PRISM task [28,30]. In their PRISM-R and PRISM-R2 tasks, before completing the basic PRISM task, informants are asked to choose their ‘Illness’ disk from three offered, one smaller than the Self disk, one the same size, and the third larger than the Self. The size of disk chosen reflects what the researchers termed the Illness Perception Measure [28].

Two studies have replaced the fixed and moveable disks of the PRISM board with coins [31,32]. In the former study, the coins were placed on an A4 sheet of paper, evidently imitating the original PRISM task. In the latter study, details of the task were imprecise.

In most studies, the PRISM instrument is a physical object. However, some studies have adapted PRISM for computer administration [33,34]. PRISM and PRISM+ are now available as an iPad and iPhone App (called iPRISM). A recent publication has reported that iPRISM is acceptable to respondents, and appears to perform in the same way as the original PRISM task [35].

### Using PRISM

Most studies have been carried out with adults, including older adults [36]. One study focused on children aged 5–16 years [37]. in this study, the informants were asked to use PRISM and complete a quality of life questionnaire. The youngest children preferred PRISM, but the researchers considered their responses unreliable. Based on their findings, the authors of this paper suggested that PRISM was probably unsuitable for children younger than age 12 years. However, we are aware of PRISM being used successfully with children as young as 8 years old (see below).

Instructions for the PRISM task are brief and simple. Only a small minority of informants have failed to understand the basic instructions [4,11,13], and in such cases, where PRISM is administered in the presence of a researcher or clinician, the task has become clear through the use of an example (see above). In the original study [2], 2 of 26 patients needed the example, but this may have reflected the inexperience of the researchers at the time. The growing use of PRISM in self-administered form (see above) confirms that informants can follow the instructions.

A key feature of PRISM is that when informants have placed their disks on the PRISM board, if they are then asked to explain why they placed the disks as they did, they can usually offer a succinct explanation (see below).

Test-retest reliability of the task has been very satisfactory at intervals of a few hours (r = 0.95) [4], one day (r = 0.98) [15] and three days (ICC = 0.99) [13]. Inter-rater reliability has also been satisfactory (r = 0.79) [4].

### Results using PRISM to assess suffering

Discussion of the published results inevitably concentrates on PRISM as a measure of suffering, because this has been by far the most frequent application of PRISM to date.

Qualitative data from the initial studies indicated that, despite the simple instructions for PRISM, informants appeared consistent in their understanding of the task. In general, the closer the Illness disk was placed to the Self, the more difficult informants perceived their symptoms were to control, and the more their illness intruded in their lives [4]. The same themes have emerged in later qualitative studies [18,38].

Quantitatively, the most consistent significant negative correlations of Self-Illness Separation (SIS) have been with depression [2,4,13,16,22,23,26,39,40], and with pain [2,4,24] particularly appraisal of pain [15]. Studies have also reported significant negative correlations between SIS and disease-specific or generic health-related quality of life measures [22,26,41–44]. However, some contradictory findings have been reported. Streffer *et al* did not find the expected negative correlation between SIS and depression scores, but they pointed out in their paper that depression scores were low in their sample of patients with orofacial pain, and their finding might be due to a floor effect [24]. More difficult to explain are the results of Denton *et al* from their sample of patients with systemic lupus erythematosus [33]. Despite the sample selection and composition being very similar to those in another study [44], Denton *et al* failed to replicate the correlations between SIS and depression reported above, or the correlation between SIS and generic health-related quality of life (SF-36 physical component scores) reported in previous studies involving people with lupus [4,44]. Correlations between SIS and the mental or physical component subscales of the SF-36 have varied in different patient groups [4], as would be expected of a measure of suffering (see below). Significant correlations have been reported in some studies [15] but not in others [2].

Where PRISM has been used in intervention studies, it has been found to be sensitive to change. As expected, SIS increased with favourable outcomes [4,40,41,45]. Following an initial report of the therapeutic potential of PRISM [3], a later small case series used PRISM as a tool in shared decision-making between clinician and patient. The use of PRISM led to greater improvement than a standard clinical interview in patients’ perceived capacities to understand and work on their problems [21]. The study by Niedermann and colleagues [19,20] is noteworthy because, to our knowledge, it is the first randomised controlled trial to use PRISM therapeutically. A sample of people with rheumatoid arthritis was randomised to receive a joint protection education intervention which was either PRISM-based, or conventional. Here, PRISM was used not to assess suffering or the burden of the illness, but to identify individual strengths and resources, and as an aid in learning about and managing joint protection. Both groups improved in their joint protection behaviours, with the PRISM-based intervention group having significantly better specific outcomes than the conventional intervention.

Studies using the two-step revised PRISM task (PRISM-R and PRISM-R2) have reported few significant correlations with SIS. In the original publication, the initial variable elicited by the task (the Illness Perception Measure) correlated significantly with health status and wellbeing, but correlations with SIS were not significant [28]. Although studies have reported correlations between SIS and the Illness Perception Measure [34,36], correlations with other variables have been greater with the Illness Perception Measure than with SIS [30].

Denton and colleagues applied PRISM in the standard way, but reported results that were inconsistent with those from other studies (see above)[33]. This group interpreted PRISM as not measuring suffering but rather a person’s enmeshment with his or her illness. In a later studies of people with epilepsy, they reported no significant correlation between ‘enmeshment’ and either depression or suicide risk [46], but a negative correlation between ‘enmeshment’ and anxiety [47].

### Other applications of PRISM

Reinhardt and colleagues applied PRISM to people screened in primary care as problem drinkers [27]. Whereas the studies of suffering (above) asked people to indicate where their illness was in their life, in this study informants were asked to indicate where they placed alcohol in their lives. In this study, SIS correlated with readiness for change according to the Stages of Change model. Separation between ‘Self’ and ‘Alcohol’ was greater among people who were not yet ready to consider changing their drinking (that is, in the pre-contemplation stage) than those motivated and intending to change their drinking (in the action stage).

Haas and colleagues modified PRISM to assess suicidal intent, replacing the ‘illness’ label in the original task with ‘your present desire to take your own life’ [48,49]. This so-called PRISM-S task was easily understood, and accepted well by patients and the clinical team. The Self-Suicidality Separation correlated significantly with a validated self-administered suicidality scale and with depression, and qualitative data indicated that patients understood the task in a consistent manner.

In another study of people with pancreatic cancers, PRISM was used to measure perceived social support [50]. The authors reported a significant, though modest, correlation between social support measured by PRISM and that derived from a previously validated instrument. However, there was insufficient detail in the paper to evaluate precisely how PRISM was used.

Two studies have used PRISM with people who had been subjected to political turmoil and/or had their human rights violated. In one study [31], the standard ‘illness’ disk was labelled ‘political events’, and the distance between this and the Self disk was considered a measure of how involved informants were in their daily lives with political events. The authors noted that they chose to use PRISM because of its simplicity, minimising problems of language and translation. Among informants exposed to terror, those who showed small separations between the disks had significantly more symptoms of post-traumatic stress disorder than those with larger separations. In the other study [17], PRISM was used with victims of human rights violations to assess informants’ closeness to their own cultural group in relation to the cultural group allegedly involved in perpetrating the violations. A separate disk was used for each cultural group. Closeness to the informants’ own cultural group was measured as the ratio of separations from the Self of the disks representing their own versus the other cultural group. Closeness to one’s cultural group measured in this way was found to be a significant negative predictor of readiness for reconciliation.

Recently, PRISM was used to measure the perceptions among travellers to tropical and subtropical countries of various risks eg malaria, endemic outbreaks, adverse effects of vaccination etc., and to compare travellers’ appraisals before and after travelling with those of tropical medicine experts [51]. In the PRISM task, smaller separations between Self and an identified risk were interpreted as indicating greater salience of that risk. The authors reported that for some risks, travellers’ appraisals changed after travelling. Also, the experts’ risk appraisals captured by PRISM were consistent with evidence from the published literature, supporting the validity of the PRISM data.

In another study, PRISM was used in an assessment of work-related stress among anaesthesiologists [32]. In addition to the usual ‘Self’ disk, another disk was labelled ‘Work’. The authors argued that greater distance between these two disks reflected the extent of the informant’s mental detachment from work. They proposed that the combination of high perceived work stress and greater detachment probably reflected burnout, and reported that this combination of factors was associated with elevated levels of waking cortisol.

## Discussion

### The PRISM task

The PRISM task is easily understood and can be self-administered. It can be completed very rapidly. Despite its simplicity, the quantitative results show good test-retest reliability. Informants, perhaps with the exception of some teenagers [37], engage well with the task, and appear to find it more interesting than completing a questionnaire. The availability of PRISM as an iPad and iPhone App is likely to make it more attractive to young people. PRISM yields personally salient data (see also below).

Published results to date do not provide any reason to modify the task substantially. If, as is argued below, PRISM functions as a visual metaphor, making the task more complicated is likely to interfere with its effectiveness. This is the most likely reason why studies using the two-step PRISM-R and PRISM-R2 have yielded results which are different from those with the standard PRISM task. PRISM-R2 has clearly yielded interesting data, but the second stage of the task, which should be equivalent to the standard PRISM task, probably measures something different from the original method.

It might be tempting to replace the disks used in the task with icons representing the objects being investigated. However, in eliciting relationships there may be an advantage to using disks of a similar shape, and using dissimilar shapes might detract from the task [52]. For the same reason, using coins in place of the PRISM disks might complicate the task, and risks influencing informants’ responses. It remains to be tested whether giving informants the option of choosing the colour of their Illness disk [45] interferes with or enhances the task.

The standard PRISM task uses a rectangular or square PRISM board. It may seem more logical or more appealing to have a circular board to represent ‘life at the moment’ and a central disk to represent ‘Self’. However, the standard design works more effectively in allowing greater movement of the Illness disk. Also, when using PRISM+, the standard design forces informants to review the relationships between the disks, since they cannot all be placed in a circle equidistant from the Self, but their distances from the Self must be prioritised.

There has been no published reference to date on the potential significance of the angle at which the Illness disk is placed, relative to the long side of the rectangular PRISM board. Data as yet unpublished indicate that people who place the Illness disk near the short axis of the PRISM board have higher depression scores and lower SF-36 health-related quality of life scores than those who place the Illness disk along the diagonal axis, regardless of the actual Self-Illness Separation. This is consistent with depressed people, and those with a greater symptom burden, being less optimistic than others about the extent to which the Illness can ‘move away’ from the Self. This also contributes to answering the question whether PRISM is simply a complicated visual analogue scale, as has been implied [9]. Visual analogue scales are customarily anchored at both ends. This does not apply to PRISM, and certainly not to PRISM+.

### PRISM as a measure of suffering

There is no ‘gold standard’ measure of suffering against which PRISM can be validated. However, a recent systematic review [8] identified ten measures of suffering for use in palliative care, and PRISM was scored as superior to other measures for its overall psychometric properties. The reviewers also noted PRISM to be ‘the most conceptually coherent instrument’ to measure suffering.

A consistent finding from published studies, as noted above, is that Self-Illness Separation (SIS), the quantitative measure derived from PRISM, behaves as would be predicted by Cassell’s conceptualisation of suffering [5,14]. Summarising the results presented above, SIS has shown statistically significant correlations with other measures of the experience of illness, but the strength of these correlations has varied according to diagnosis and also between samples with the same diagnosis. In essence, this is exactly what would be expected of a measure of suffering, as defined by Cassell [5,14], as a perceived severe threat to an individual’s Personhood [4]. Thus, for example, fatigue is likely to represent a more severe and salient threat to Personhood in cancer survivors who experience severe fatigue, than among people with rheumatoid arthritis, where pain and/or stiffness are likely to have a greater influence. In conditions where symptoms or handicaps constitute a major threat to Personhood, SIS would be expected to correlate with disease-specific symptom inventories, or possibly with more generic measures such as the SF-36.

How a person appraises the threat from an illness to his or her Personhood is complex, and involves factors beyond the burden of symptoms or the extent of disabilities. This is illustrated by the inverse correlation of suffering, measured by Self-Illness Separation, and sense of coherence as a measure of personal resilience against stress [4,53]. There is considerable evidence that, independent of illness severity, a high sense of coherence is associated with less depression [53] and better health outcomes [54]. While some studies have reported no relationship between SIS and age [22,27], others have reported correlations with age [44] or time since diagnosis [12]. If, as above, suffering results from serious threats to Personhood, changes to a person’s appraisal of Personhood would be expected to result in changes in suffering. With some conditions, longer time since diagnosis might lead a person to reappraise his or her Personhood–essentially reviewing what is important in one’s life–hence offering scope to also alter suffering [55]. Ageing could lead a person to relinquish previously valued aspects of Personhood, this again working to reduce suffering. On the other hand, if someone has already experienced a ‘narrowing’ of Personhood through persistent symptoms and/or disability, the threat of losing even more of one’s Personhood could lead to greater suffering.

### Generic conceptualisation of the PRISM task

PRISM was not developed from first principles according to any theoretical model. To date, despite the consistent results in applying PRISM to measure suffering (or perhaps because of this), little attempt has been made to understand how PRISM works. This has become the more important because, as noted above, in novel applications other than measuring suffering, PRISM has also yielded expected and statistically significant correlations with other measures. How is it possible that a valid measure of suffering can also measure the perceptions of risks by tourists and work-related stress among doctors? Reflecting on all the published studies to date, including the novel applications, allows a generic conceptualisation of PRISM’s mechanism of action.

**Fig 2:**
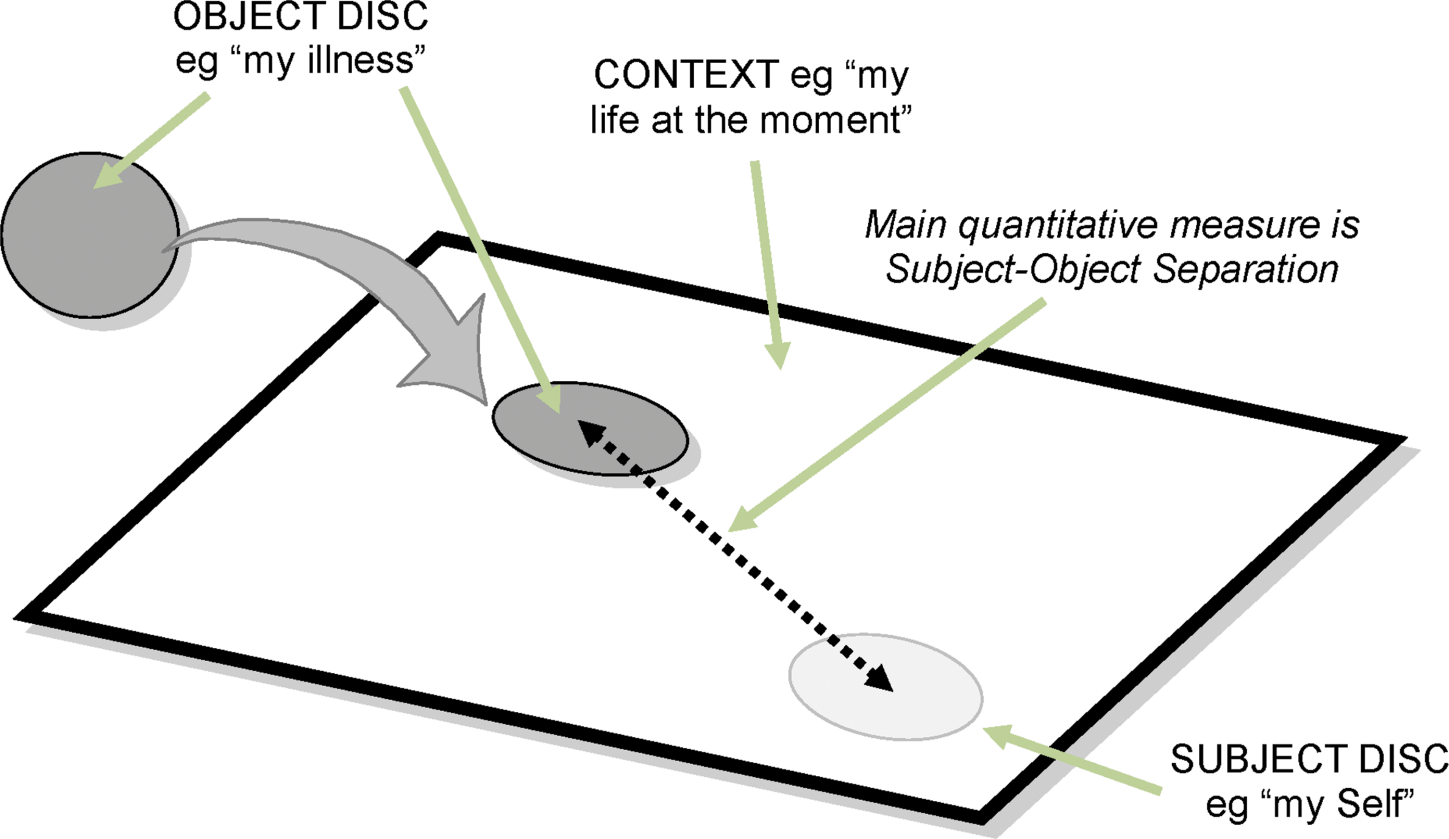
The PRISM instrument.

All the results to date, including the newer applications of PRISM as well as the studies of suffering, indicate that PRISM owes its success to the fact that it functions as a visual metaphor (see below), measuring the strength of the relationship between a Subject and Object(s) putatively related to that Subject, within a defined context (Fig 2). Applying PRISM to the measurement of suffering, the Subject is ‘my Self’, the Object is ‘my illness’, and the context is ‘my life at the moment’.

The basis of PRISM’s functioning as a visual metaphor can be explained by the observation that in placing concepts or ideas on paper or screen, the proximity between features is determined by the strength of the associations between them. This is termed the distance-similarity metaphor [56], and is also consistent with the gestalt principle of proximity, which states that elements that are closer together are seen as a group and perceived as more closely related than other elements in the image or display. Where the Subject is ‘Myself’, Objects which are more salient to the person within the defined context (for example, my life as it is now), are placed closer to the Self. Note that the putative relationship between subject and object does not need to be a positive one; ‘my illness’ has a negative relationship with ‘my Self’, and the closer the illness is to the Self, the more negative its effects, in terms of intrusiveness in everyday life, and lack of controllability. Similarly in the study by Zimmerman and colleagues [51], risks perceived by travellers as particularly salient for forthcoming trips were placed closer to the Self. Spatial metaphors form a conceptual scaffolding between concepts [57]. The role of this conceptual scaffolding is to create associations between ideas or concepts, which are then elaborated in a personally salient way. Even the most abstract states can be viewed as physical locations and changes in those states can be viewed as movements between the equivalent locations.

A key point about all metaphors is that they are deliberately vague. To interpret a metaphor, a person cannot rely on what has been presented, but must reach a specific understanding of the metaphor, which inevitably requires interpretations which are personally salient. How informants respond to PRISM can be understood in terms of the Relevance Theory of Metaphor [58]. In essence, this theory suggests that people approach metaphors by testing their own hypotheses regarding their meaning, and conclude their interpretation when they reach an understanding which is personally relevant and meaningful. Interpreting metaphors involves conceptualising vague, abstract domains of knowledge in terms of more specific, familiar, concrete personal knowledge or experience. Metaphors can only be understood in a personal context and in a personally salient way [59]. The fact that PRISM can measure suffering (or other Subject-Object relationships) indicates that even personally salient details often follow particular themes. Although each person’s experience of illness and suffering is unique, and this uniqueness is reflected in how he/she responds to the PRISM task, important themes emerge from quantitative and qualitative analyses of the data. Most notable is that the distance between Illness and Self correlates inversely with intrusiveness and lack of controllability of the illness [4,18]. As a visual metaphor, PRISM forces informants to interpret the task in a personally salient way. As noted above, informants are usually able to describe very succinctly their personally salient beliefs or appraisals on completion of the task, when asked to do so. Asking someone directly: ‘What causes you to suffer in your life at the moment?’ is likely to result in a lengthy narrative response, with the informant having to define his or her life at the moment, note aspects of life that he or she particularly valued, and how these had been affected by the illness. By contrast, using PRISM and asking ‘Why did you put the Illness disk there?’ usually elicits a brief but focused and pertinent response.

### Strengths and potential limitations of PRISM

Some of the strengths of PRISM have already been outlined above. It is simple to administer, readily understood, and yields personally salient results quickly. Unlike interviews or questionnaires, it is very unlikely that informants will guess responses which are either socially desirable or which they think might be favoured by the researcher or clinician. In this context, it is interesting that in the study by Reinhardt *et al* of people with alcohol problems, “alcohol” moved closer to the Self as patients became more motivated to recognise and manage their alcohol use [27]. This can be seen as part of taking ownership of the problem, which is essential in motivating change. However, it is very unlikely that patients could have worked this out themselves before completing the PRISM task. Experience in the use of PRISM also indicates that informants identify with the task and their responses much more directly than with questionnaires they have completed. One possible reason for this is that PRISM leads to the generation of the informant’s own personal metaphor. Seeing a novel task as personally salient could increase the processing resources a person devotes to the task [60].

There are also more general advantages to using visual rather than verbal processes. In a paper aptly titled ‘why a diagram is (sometimes) worth ten thousand words’, it was argued that finding new relationships and problem-solving is facilitated by diagrammatic as opposed to verbal presentations [61]. Verbal information is organised linearly, and relationships need to be described, while visual representations show relationships, and support a larger number and range of perceptual inferences. Presenting information visually can encourage recall [62]. The ambiguity of PRISM as a visual metaphor encourages flexibility in thinking, and the possibility of divergent interpretations [63], both of which are also likely to facilitate creative thinking. This can be a distinct advantage, but not always. As with other visualisations, PRISM constitutes a form of representational guidance. It acts as a representational tool allowing participants to construct and appraise external representations of their knowledge [64].

PRISM only works well when care has been taken to define appropriately the Subject, Object and Context under investigation. Because it works as a metaphor, there must be sufficient flexibility in the definitions to allow personally salient interpretations of the task. The results summarised above indicate that the definitions used to assess suffering work well. However, new applications of PRISM probably need to be piloted to develop and refine the optimal instructions for the task. If the definitions and instructions are too specific, the value of the metaphor may be lost. On the other hand, defining the parameters too vaguely makes the task harder to understand and risks yielding inconsistent results. Changing metaphors can lead to informants thinking differently and reaching different conclusions [65], which is likely to account for the differences found in results from the original PRISM task and the revised PRISM-R and PRISM-R2 tasks (see above). An important limitation in using PRISM to gather quantitative data is that the relationship between Subject and Object must be univalent (that is, either positive or negative). A person’s illness or symptoms are almost universally regarded as negative, thus the closer the illness is to the Self disk, the more negative the person’s view or appraisal. However if the Object is ‘my partner’, putting the ‘Partner’ disk close to the self could indicate a close and loving relationship, but it could also reflect that the partner is domineering or intrusive. In this context, it is interesting that Klein and colleagues were successful in applying PRISM to measure the ‘mental distance’ between anesthesiologists and their work [32]. Work is not usually univalent, and putting work close to the self could indicate that the person enjoys her work rather than finding it intrusive. However, it may be that the participants in this study were all influenced by a particularly difficult shared work environment, noted as a reason for undertaking the study.

PRISM is likely to appeal to young people because of its interactive, game-like features and its availability on the iPad, but it remains unclear at what age children might be expected to respond consistently to the PRISM task. Melbardis Jorgensen and Jemec concluded that children under 12 years old gave inconsistent responses to PRISM [37], but it remains unclear whether this was because they lacked the capacity to respond to the task, or because the instructions for the task were not optimised. These authors offered an explanation for this specific result in terms of the Piagetian model of child development. Others have offered similar interpretations of children’s understanding of metaphor [66]. On the other hand, it is widely recognised that children much younger than 12 years use metaphors spontaneously in their speech, and can also understand some metaphors. This apparent contradiction can be reconciled by considering how children understand different types of metaphor. Attributional metaphors are based on the elements sharing object attributes eg the sun is an orange (both are round and have a similar colour). Relational metaphors are based on the elements sharing a common relational structure. For example, it would be difficult to interpret ‘Jennifer is a fish’ in terms of structural similarities between Jennifer and a fish, but both elements might share relations, such as being good at swimming. There is evidence that children’s abilities to understand attributional metaphors change little after the age of 5–6 years, while understanding of relational metaphors increases with age [67]. This is not simply a function of cognitive development; the ability to understand metaphors also depends on familiarity with the elements of the metaphor, which may depend more on experience than on cognitive ability [68]. All these factors need to be considered in PRISM applications with children, and again highlight the importance of pilot work.

To date, PRISM has mainly been used in studies in Europe and North America. However, evidently successful applications of PRISM have been reported from studies in Colombia [12], Brazil [13], Nepal [31],the Gambia [39], and among Kurdish refugees [17]. This suggests that the distance-similarity metaphor which forms the basis of PRISM is likely to be understood similarly in different cultures. However, cross-cultural comparisons might be complicated by different understanding between cultures of the chosen Subject or Objects. For example, ‘Self’ may be understood differently in different cultures [69]. There is also evidence that perceptual processes may differ between cultures, with people from Eastern cultures paying more attention to context than those from Western cultures [70].

## Conclusion

A 2009 editorial commenting on the use of PRISM in dermatology [9] was sceptical, commenting ‘who knows what the parabola [the space encompassing the disks] means for each individual, and who knows what this distance means?’. Given the novelty and simplicity of PRISM, such scepticism is to be expected. However, the correlations between PRISM and other validated measures, and the degree of their consistency across studies, indicate that informants interpret the PRISM task in a reliable and consistent way. Understanding PRISM as a visual metaphor offers a plausible and coherent explanation of how PRISM works.

The consistent results of PRISM as a measure of suffering suggest that it has the potential in healthcare and social science to further elucidate the nature and associations of suffering. One recent example is a report distinguishing the presence of tinnitus from suffering associated with it, which identified different brain areas associated with each of these [26]. Improved understanding of the nature of suffering, together with a simple means of measuring it, opens the possibility of refining interventions aimed at alleviating it. PRISM can serve as an aid to communication between patients and their clinicians [3], by eliciting salient aspects of a person’s illness experience and enhancing person-centred medicine and shared decision-making. It could be used in training medical students and other health professionals to approach their patients in a person-centred and empathic way. In its present form, PRISM can already be used to compare patients’ appraisals of the relative burden of two different illnesses or symptoms. In clinical practice, it has been used with individual patients as a person-centred outcome measure [21] (that is, a patient-reported outcome measure). With attention to the instructions for the task, this can be extended for use in therapeutic trials, where the simplicity and brevity of PRISM have distinct advantages.

Studies using PRISM in settings other than healthcare, such as the recent study by Zimmerman and colleagues of appraisal of hazards by travellers and experts [51] and similar studies currently underway [71], illustrate how PRISM can readily be adapted to explore a variety of questions about appraisal, attitudes or beliefs, or decision-making. For example, if the Subject is ‘the perfect candidate for this post’, and the Objects are individual qualities or competencies, PRISM could be used to achieve a consensus on competencies required for that particular appointment. Asking an offender where in his life the victim is at the moment could yield information about his readiness to accept responsibility for his crime. The board of a company could use PRISM to examine the consistency among its members regarding priorities, one example of visual strategizing [72]. At the individual level, PRISM is likely to be helpful in coaching, where its use is analogous to that in individual therapeutic interventions in psychology or medicine. PRISM lends itself to such applications because it is able to generate personally salient information, without the need for much prior discussion. Nominal group techniques were developed because it was recognised that group discussion may become biased for a variety of reasons and under some circumstances can be unhelpful [73,74]. PRISM could be used to facilitate nominal group technique procedures, or alongside standard nominal group processes, as well as other methods of reaching consensus, such as the RAND/UCLA appropriateness method [75]. Technologies are being developed to use PRISM in groups and get feedback in real time [76]. Use of PRISM in this way would be an example of collaborative visualisation, which has the potential to access, share and use more knowledge among informants than group discussions, and also facilitates productive participation by all group members [77].

PRISM was developed initially from a serendipitous observation, as noted above. It seems unlikely that it could have been developed from first principles. However, published results from its use as well as existing literature on metaphor, visualisation and in other diverse fields support the proposed conceptualisation, and this in turn serves to identify a growing range of potential applications.

## Supporting Information

S1 Appendix Bibliography of papers reported data using PRISM. (DOCX)

S1 PRISMA 2009 Checklist (DOC)

S1 PRISMA Checklist Notes (DOC)
