# Supplementary material for: PRISM, a Novel Visual Metaphor Measuring Personally Salient Appraisals, Attitudes and Decision-Making: Qualitative Evidence Synthesis
Source: PLoS One. 2016 May 23;11(5):e0156284. doi: 10.1371/journal.pone.0156284 (PMC4877057; doi:10.1371/journal.pone.0156284)
Supplement: S1 Appendix — (DOCX) [file pone.0156284.s001.docx]

**S1 APPENDIX: Bibliography of papers reported data using PRISM**

Buchi S, Sensky T, Sharpe L, Timberlake N (1998) Graphic representation of illness: a novel method of measuring patients' perceptions of the impact of illness. Psychother Psychosom 67: 222-225.

Buchi S, Sensky T (1999) PRISM: Pictorial representation of illness and self measure: A brief nonverbal measure of illness impact and therapeutic aid in psychosomatic medicine. Psychosomatics 40: 314-320.

Buchi S, Brandli O, Klingler K, Klaghofer R, Buddeberg C (2000) [Inpatient rehabilitation in inpatients with chronic obstructive lung diseases (COPD): effect on physical capacity for work, psychological wellbeing and quality of life]. Schweiz Med Wochenschr 130: 135-142.

Buchi S, Villiger P, Kauer Y, Klaghofer R, Sensky T, Stoll T (2000) PRISM (Pictorial Representation of Illness and Self Measure)- a novel visual method to assess the global burden of illness in patients with systemic lupus erythematosus. Lupus 9: 368-373.

Buchi S, Buddeberg C, Klaghofer R, Russi EW, Brandli O, Schlosser C, Stoll T, Villiger PM, Sensky T (2002) Preliminary validation of PRISM (Pictorial Representation of Illness and Self Measure) - a brief method to assess suffering. Psychother Psychosom 71: 333-341.

Buchi S, Morgeli H, Schnyder U, Jenewein J, Hepp U, Jina E, Neuhaus R, Fauchere JC, Bucher HU, Sensky T (2007) Grief and post-traumatic growth in parents 2-6 years after the death of their extremely premature baby. Psychother Psychosom 76: 106-114.

Buchi S, Morgeli H, Schnyder U, Jenewein J, Glaser A, Fauchere JC, Ulrich BH, Sensky T (2009) Shared or discordant grief in couples 2-6 years after the death of their premature baby: effects on suffering and posttraumatic growth. Psychosomatics 50: 123-130.

Buchi S, Straub S, Schwager U (2010) [Shared Decision-Making and Individualized Goal Setting - a Pilot Trial Using PRISM (Pictorial Representation of Illness and Self Measure) in Psychiatric Inpatients.]. Praxis 99: 1467-1477.

Ciaccio S, Girelli A, Rocca L, Cimino A, Valentini U (2003) [PRISM: a method to visualize the subjective burden of the illness]. Gionale Italiano de Diabetologia e Metabolismo 23: 189-194.

Denton F, Sharpe L, Schrieber L (2004) PRISM: Enmeshment of illness and self-schema. Psychother Psychosom 73: 57-63.

Duncan E, Gidron Y, Shrestha RP, Aryal T (2005) Correlates of post-traumatic stress and physical symptoms in Nepali adults under political turmoil. Australasian Journal of Disaster and Trauma Studies. http://researchonline.gcu.ac.uk/portal/en/publications/correlates-of-posttraumatic-stress-and-physical-symptoms-in-nepali-adults-under-political-turmoil%28ab6d20b1-9d97-42f8-9728-337ea74bd7f9%29.html

Gandy M, Sharpe L, Perry KN, Miller L, Thayer Z, Boserio J, Mohamed A (2013) The psychosocial correlates of depressive disorders and suicide risk in people with epilepsy. J Psychosom Res 74: 227-232.

Gandy M, Sharpe L, Perry KN, Miller L, Thayer Z, Boserio J, Mohamed A (2015) Anxiety in epilepsy: a neglected disorder. J Psychosom Res 78: 149-155.

Gardner-Nix J, Barbati J, Grummitt J, Pukal S, Raponi Newton S (2012) Exploring the effectiveness of a mindfulness-based chronic pain manaement course delivered simultaneously to on-site and off-site patients using telemedicine. Mindfulness doi: 10.1007/s12671-012-0169-3.

Gielissen MF, Prins JB, Knoop H, Verhagen S, Bleijenberg G (2013) Pictorial Representation of Self and Illness Measure (PRISM): a graphic instrument to assess suffering in fatigued cancer survivors. Psychol Assess 25: 658-663.

Gois CJ, Ferro AC, Santos AL, Sousa FP, Ouakinin SR, Do Carmo I, Barbosa AF (2012) Psychological adjustment to diabetes mellitus: Highlighting self-integration and self-regulation. Acta Diabetol 49: S33-S40.

Harbauer G, Ring M, Schuetz C, Andreae A, Haas S (2012) Suicidality Assessment with PRISM-S - Simple, Fast, and Visual. Crisis 34: 1-6.

Hoffenkamp HN, Tooten A, Hall RA, Croon MA, Braeken J, Winkel FW, Vingerhoets AJ, van Bakel HJ (2012) The impact of premature childbirth on parental bonding. Evol Psychol 10: 542-561.

Kassardjian CD, Gardner-Nix J, Dupak K, Barbati J, Lam-McCullock J (2008) Validating PRISM (Pictorial Representation of Illness and Self Measure) as a measure of suffering in chronic non-cancer pain patients. J Pain 9: 1135-1143.

Klein M, Weksler N, Gidron Y, Heldman E, Gurski E, Smith OR, Gurman GM (2012) Do waking salivary cortisol levels correlate with anesthesiologist's job involvement? J Clin Monit Comput 26: 407-413.

Klis S, Vingerhoets AJ, de WM, Zandbelt N, Snoek FJ (2008) Pictorial Representation of Illness and Self Measure Revised II (PRISM-RII): a novel method to assess perceived burden of illness in diabetes patients. Health Qual Life Outcomes 6: 104. doi:10.1186/1477-7525-6-104.

Klis S, Velding K, Gidron Y, Peterson K (2011) Posttraumatic stress and depressive symptoms among people living with HIV in the Gambia. Aids Care 23: 426-434.

Knopfli BH, Radtke T, Lehmann M, Schatzle B, Eisenblatter J, Gachnang A, Wiederkehr P, Hammer J, Brooks-Wildhaber J (2008) Effects of a multidisciplinary inpatient intervention on body composition, aerobic fitness, and quality of life in severely obese girls and boys. J Adolesc Health 42: 119-127.

Krikorian A, Limonero JT, Roman JP, Vargas JJ, Palacio C (2013) Predictors of Suffering in Advanced Cancer. Am J Hosp Palliat Care 31: 534-542.

Krikorian A, Limonero JT, Vargas JJ, Palacio C (2013) Assessing suffering in advanced cancer patients using Pictorial Representation of Illness and Self-Measure (PRISM), preliminary validation of the Spanish version in a Latin American population. Support Care Cancer 21: 3327-3336.

Lehmann V, Oerlemans S, van de Poll-Franse LV, Vingerhoets AJ, Mols F (2011) Suffering in long-term cancer survivors: an evaluation of the PRISM-R2 in a population-based cohort. Qual Life Res 20: 1645-1654.

Lima-Verde AC, Pozza DH, Rodrigues LL, Velly AM, Guimaraes AS (2013) Cross-cultural adaptation and validation for Portuguese (Brazilian) of the pictorial representation of illness and self measure instrument in orofacial pain patients. J Orofac Pain 27: 271-275.

Melbardis Jorgensen K, Jemec GBE (2011) Evaluating PRISM (Pictorial Representation of Illness and Self Measure) as a measure of life quality for children with skin diseases. Eur J Pediatric Dermatol 21: 135-142.

Meyer M, Luethi MS, Neff P, Langer N, Buchi S (2014) Disentangling tinnitus distress and tinnitus presence by means of EEG power analysis. Neural Plast 2014: Article ID 468546 doi: 10.1155/2014/468546

Muhleisen B, Buchi S, Schmidhauser S, Jenewein J, French LE, Hofbauer GL (2009) Pictorial representation of illness and self measure (PRISM): A novel visual instrument to measure quality of life in dermatological inpatients. Arch Dermatol 145: 774-780.

Niedermann K, de Bie RA, Kubli R, Ciurea A, Steurer-Stey C, Villiger PM, Buchi S (2011) Effectiveness of individual resource-oriented joint protection education in people with rheumatoid arthritis. A randomized controlled trial. Patient Educ Couns 82: 42-48.

Niedermann K, Buchi S, Ciurea A, Kubli R, Steurer-Stey C, Villiger PM, de Bie RA (2012) Six and 12 months' effects of individual joint protection education in people with rheumatoid arthritis: a randomized controlled trial. Scand J Occup Ther 19: 360-369.

Rajpura J, Nayak R (2014) Medication adherence in a sample of elderly suffering from hypertension: evaluating the influence of illness perceptions, treatment beliefs, and illness burden. J Manag Care Pharm 20: 58-65.

Reimus JL, Vingerhoets AJ, Soons PH, Korstanje MJ (2007) Suffering in psoriasis patients: its relation with illness severity and subjective well-being. Int J Dermatol 46: 1042-1045.

Reinhardt S, Bischof G, Grothues J, John U, Rumpf HJ (2006) Performance of the Pictorial Representation of Illness and Self Measure in individuals with alcohol dependence, alcohol abuse or at-risk drinking. Psychother Psychosom 75: 249-256.

Ring M, Harbauer G, Haas S, Schuetz C, Andreae A, Maercker A, Ajdacic-Gross V (2014) [Validity of the suicidality assessment instrument PRISM-S (Pictoral Representation of Illness Self Measure - Suicidality)]. Neuropsychiatr 28: 192-197.

Rumpf HJ, Lontz W, Uesseler S (2004) A self-administered version of a brief measure of suffering: first aspects of validity. Psychother Psychosom 73: 53-56.

Schmid-Buchi S, Halfens RJ, Dassen T, van den Borne B (2011) Psychosocial problems and needs of posttreatment patients with breast cancer and their relatives. Eur J Oncol Nurs 15: 260-266.

Stammel N, Neuner F, Bottche M, Knaevelsrud C (2012) Construction of a questionnaire for readiness to reconcile in victims of human rights violations. Eur J Psychotraumatol 3: Article ID 15785 doi: 10.3402/ejpt.v3i0.15785.

Stefaniak TJ, Dziedziul J, Walerzak AM, Stadnyk M, Sheikh A, Proczko-Markuszewska M, Laski D, Vingerhoets AJJM, Zadrozny DK, Smietanska IA, Lachinski AJ (2012) Pain intensity and perceived social support among patients with pancreatic tumors. J Pain Relief 1:110. doi:10.4172/2167-0846.1000110.

Streffer ML, Buchi S, Morgeli H, Galli U, Ettlin D (2009) PRISM (pictorial representation of illness and self measure): a novel visual instrument to assess pain and suffering in orofacial pain patients. J Orofac Pain 23: 140-146.

Tondury B, Muehleisen B, Ballmer-Weber BK, Hofbauer G, Schmid-Grendelmeier P, French L, Buchi S (2011) The Pictorial Representation of Illness and Self Measure (PRISM) instrument reveals a high burden of suffering in patients with chronic urticaria. J Investig Allergol Clin Immunol 21: 93-100.

van Bakel HJ, Maas AJ, Vreeswijk CM, Vingerhoets AJ (2013) Pictorial representation of attachment: measuring the parent-fetus relationship in expectant mothers and fathers. BMC Pregnancy Childbirth 13: 138. doi: 10.1186/1471-2393-13-138.

Weidt S, Bruehl AB, Moergeli H, Straumann D, Hegemann SCA, Buchi S, Rufer M (2015) Graphic representation of the burden of suffering in dizziness patients. Health Qual Life Outcomes 12: 184. doi: 10.1186/s12955-014-0184-2

Wittmann L, Buchi S (2010) Personal transformation in chronic physical disease: conceptual limitations of the posttraumatic growth construct. Psihologija 43: 375-388.

Wittmann L, Schnyder U, Buchi S (2012) PRISM (Pictorial Representation of Illness and Self Measure): a new method for the assessment of suffering after trauma. J Trauma Stress 25: 94-97.

Wittmann L, Sensky T, Meder L, Michel B, Stoll T, Buchi S (2009) Suffering and Posttraumatic Growth in Women With Systemic Lupus Erythematosus (SLE): A Qualitative/Quantitative Case Study. Psychosomatics 50: 362-374.

Wouters EJ, Reimus JL, van Nunen AM, Blokhorst MG, Vingerhoets AJ (2008) Suffering quantified? Feasibility and psychometric characteristics of 2 revised versions of the Pictorial Representation of Illness and Self Measure (PRISM). Behav Med 34: 65-78.

Wouters EJ, van Nunen AM, Vingerhoets AJ, Geenen R (2009) Setting overweight adults in motion: the role of health beliefs. Obes Facts 2: 362-369.

Wouters E, Geenen R, Vingerhoets A (2009) Suffering and quality of life predict dropout from physical exercise programs in obese adults. Psychologie en Gezondheid 37: 267-275.

Zimmermann R, Hattendorf J, Blum J, Nuesch R, Hatz C (2013) Risk perception of travelers to tropical and subtropical countries visiting a swiss travel health center. J Travel Med 20: 3-10.
