# Supplementary material for: PRISM, a Novel Visual Metaphor Measuring Personally Salient Appraisals, Attitudes and Decision-Making: Qualitative Evidence Synthesis
Source: PLoS One. 2016 May 23;11(5):e0156284. doi: 10.1371/journal.pone.0156284 (PMC4877057; doi:10.1371/journal.pone.0156284)
Supplement: S1 PRISMA Checklist Notes — (DOC) [file pone.0156284.s003.doc]

**S2 PRISMA Checklist Notes**

Item 1: The present manuscript fulfils the Cochrane Collaboration definition of a systematic review (cited in Moher D et al, PLOS Medicine 2009). We chose not to identify this study explicitly as a ‘systematic review’ in its title, because it is not the common form of systematic review which focuses on quantitative data. We consider that the methodology of our study is described more accurately as ‘qualitative evidence synthesis’ rather than ‘systematic review’. However, if the reviewers or editors consider that we should include ‘systematic review’ in the title, we will follow this advice.

Item 8: Papers eligible for inclusion in the study were identified by their citation of one of the original publications describing the use of the PRISM task. The details given in the manuscript (pp3-4) allow anyone else to repeat the search to yield exactly the same results, as required in PRISM checklist item 8. However, there is no ‘full electronic search strategy’ to include.

Item 14: There was no quantitative synthesis of the results. However, the key principles of qualitative evidence synthesis are outlined on p3, and also noted, with reference to this particular study, on p4.

Items 18-23: The PRISMA checklist applies particularly to systematic reviews focusing on quantitative data. For reasons that are explained in the manuscript (p4), we chose to include all publications which reported data using PRISM measure. For both these reasons, several items in the ‘Results’ section of the PRISMA checklist are therefore not applicable to this particular study.
